# Living with food allergies: the experiences of adult patients and caregivers

**DOI:** 10.3389/falgy.2023.1272851

**Published:** 2023-11-06

**Authors:** Alexandra F. Santos, Margitta Worm, Shoko Kurita, Tania Wong, Davide Contato, Elia Pirillo, A. Esther Esteban, Paolo Tassinari, Flavia Perna, R. Sharon Chinthrajah

**Affiliations:** ^1^ Faculty of Life Sciences and Medicine, King’s College London, London, United Kingdom; ^2^ Children’s Allergy Service, Evelina London Children’s Hospital, London, United Kingdom; ^3^ Division of Allergology and Dermatology, Charité, Berlin, Germany; ^4^ Japanese Mother’s Society for Allergy Care (JMSAC), Kanagawa, Japan; ^5^ Allergy & Anaphylaxis Australia, Castle Hill, NSW, Australia; ^6^ Food Allergy Italia, Padua, Italy; ^7^ Spanish Association for People with Food and Latex Allergy (AEPNAA), Madrid, Spain; ^8^ Novartis Pharma AG, Basel, Switzerland; ^9^ Sean N Parker Center for Allergy and Asthma Research, Stanford University School of Medicine, Stanford, CA, United States

**Keywords:** anaphylaxis, disease life experience, interactive dialogue, mode of action (MOA), quality of life

## Abstract

**Background:**

Few studies have addressed how food allergy may impact differently on the daily lives of adults with food allergies and caregivers for food-allergic dependents.

**Objective:**

To explore similarities and differences in life experiences and unmet needs between individuals caring for a child with food allergy and adults with food allergy world-wide.

**Methods:**

Two multinational, virtual, interactive, moderated discussions of specific questions between respectively people with food allergies and caregivers for people with food allergies, with experienced clinicians participating.

**Results:**

Sixteen individuals living with food allergies and nine caregivers took part in the two roundtables. Food avoidance and antihistamines were the most common treatments for food-allergic reactions in both groups. Caregivers reported greater burden of disease on affected individuals and families than did adult patients. Adult panelists considered autoinjectors easy to use but caregivers reported additional emotional stress thinking about autoinjector use. Caregivers described an ever-present fear of inattention and of overlooking a risk factor for a severe reaction, whereas adult panelists showed a determination not to let their food allergies interfere with living their lives. Both groups had safety-conscious attitudes to treatments, but adult patients emphasized convenience while caregivers prioritized reduced severity of reactions and eliminated fear. Both groups confirmed the need for improved, trusted sources of information, and for resources and training programs for any new therapies.

**Conclusion:**

The interactive exchange provided insights into differences between adult patients and caregivers, notably in fear and confidence in daily life, severity of disease impact, and unmet needs for treatments.

## Introduction

Food allergies affect an estimated 8% of children and 5% of adults, with greater prevalence in younger than in older children (1, 2). There is some evidence that food allergy prevalence is increasing, although the data to support this trend are mixed (2). Food is the major trigger of anaphylactic reactions in children, in contrast to adults, in whom depending on the region insect venom and drugs may occur more frequently (3, 4). Many children can become tolerant with time in case of food allergies to egg or milk, but clinical relevant sensitivities to peanuts, tree nuts or seafood appear to be more persistent (5).

Food allergies are typically managed by strict avoidance of allergens and treatment of acute allergic reactions with rescue medication (6). The disruption caused by measures taken to avoid allergen exposure has been associated with a lower quality of life (QoL) (7). The negative impact of food allergies can also manifest as anxiety and/or restriction of daily life activities. This is not limited to the affected individuals: a large body of evidence supports the powerful psychosocial impact of food hypersensitivity on caregivers and family (6–14). However, these groups experience the condition in a different context to adults with food allergy, which is likely to influence the impact. Caregivers experience the effects of allergen avoidance second-hand as primary caregivers responsible for providing nutritious allergen-free foods, and with the added burden of responsibility for children who need to learn how to manage their condition and possible reactions. By contrast, adults with food allergies are personally responsible for their management; they are often able to draw on several years of first-hand experience of reactions to food(s) and the impact of the condition on daily lives.

Several studies have explored the psychosocial impact of food hypersensitivity on patients, families and caregivers (14), and specific QoL instruments for children and adults have been developed and validated in recent years (15–17). Given the multifaceted determinants of QoL, it is of interest to explore the dynamic aspect of how views may change and develop in a dialogue with peers in the presence of healthcare providers (HCP). In addition, most studies on patient perspectives have focused on individual countries (18), which leaves the international dialogue aspect unexplored.

We report here the results of two multinational, virtual, interactive, moderated, structured discussions of specific questions between adults with food allergies or caregivers for children with food allergies, both with HCP participation. The objective was to explore the similarities and differences between the two groups in their experiences of living with, or caring for children with, food allergies. The sponsor (Novartis) had the objective to receive advice and guidance to ensure a patient-guided approach to future activities in this disease area.

## Methods

### Participants and discussion platform

The structured discussions took place in November/December 2021 (adults) and in April/May 2022 (caregivers) on a virtual advisory board platform (Within3, Lakewook, OH, USA). The interactive tool and discussion format have previously been used with similar objectives in other health conditions, as described in the literature (19, 20). Participants were recruited through their HCPs and from patient advocacy groups, with the goal of including a mix of age groups, sex, ethnicity, and geographic origin, as well as characteristics, duration, and experiences of living with or caring for people with food allergy. All food allergies had been formally diagnosed. All participants were informed on the objectives of the project and provided written, informed consent to take part in the roundtable discussion.

Participants viewed guiding questions and background presentations on food allergy within the platform, which could be accessed from any connected device at any time which suited individual schedules and time zones. Responses and comments were visible to all participants who could provide input at all stages of the discussions. Automated translation facilities enabled international participants to interact in their native languages. An independent moderator had access to all responses and could provide clarification or ask for additional information where appropriate. Two representatives of the sponsor assisted with moderation and clarification of specific issues if needed. Four allergists (RSC and MW with adults; AS and MG with caregivers) with internationally recognized expertise in food allergy represented the HCP perspectives and answered questions from the panelists. Neither HCPs nor moderators provided answers to the closed and open questions which structured the discussions.

### Discussion topics and analysis

Discussions focused on the current life experience living with food allergy or caring for children with food allergy, access to and use of information, unmet needs, and thoughts on hypothetical clinical trials in food allergy. A combination of open and closed questions were included. Examples of the former are “*How severe do you rate your/your child's food allergy to be: mild, moderate or severe?*” or “*How burdensome is living with food allergy for your family on a scale of 1–10?*” Open questions concerned matters such as “*In what ways does your food allergy impact your work and social life both physically and emotionally?*” or “*Is there anything you do differently because your child has a food allergy?*” The complete list is provided in the Supplementary Appendix. The questions were used as a basis for discussions, not as a formal survey.

All data were analyzed descriptively. As this was a qualitative study, there was no *a priori* hypothesis. Closed question results are presented numerically. Representative quotes from the participants are in italics.

## Results

Sixteen individuals living with food allergies took part in the virtual roundtable with adult patients, and nine caregivers took part in the caregiver roundtable.

### Adult patients with food allergies

Adults were aged between 18 and 47 years and lived in Australia, Canada, China, France, Germany, Italy, Japan, Spain, and the US. All except three adult participants had been living with food allergy since early childhood. Multi-food allergies were reported by 12/16 participants. Over the roundtable period of 2 weeks, a total of 1,107 posts were entered on the platform.

### Caregivers of patients with food allergy

Caregivers lived in Australia, China, Italy, Japan, Spain, and the USA; the current age of caregivers' children ranged from 4 to 19 years. Three caregivers had two children with food allergies; the others had one allergic child each. All children had presented with allergy symptoms in their first year of life, in most cases to infant formula. In the caregiver families, 6 of 12 allergic children had multi-food allergies. Four children had not outgrown any allergies with time; the others had outgrown some allergies, e.g., milk, egg or soy. Over the roundtable period, a total of 768 posts were entered on the platform.

Milk, nuts, egg and peanut were the most common allergenic foods in both roundtables. All caregivers considered their children to have “severe” allergies and 14/16 adults made the same assessment of their allergies. The most severe allergic reactions experienced by children included vomiting and exhaustion, hives, incontinence, asthma and anaphylactic shock. Adults recalled a range of symptoms from intense throat and mouth itchiness to breathing difficulties, angioedema, and loss of consciousness.

Caregivers took a pragmatic approach to assessment of severity, as reflected in the following quote:

“*As far as I am concerned, if children are limp and can't breathe from just a lick or a nibble or invisible cross contamination, that is severe!*”

### Life experience

For both roundtable groups, the initial food allergy diagnosis was most commonly made by an allergist or pediatric allergist, typically referred to by a pediatrician. Most diagnoses triggered by severe allergic reactions in childhood were made rapidly, but participants with initial mild reactions sometimes experienced a longer time to a correct diagnosis. Participants living in rural areas reported access to fewer qualified physicians, and travel was sometimes an issue when consulting specialists based in cities. Public health care was occasionally associated with delays for non-urgent consultations, and allergy-related visits to other specialists sometimes had long waiting times.

Once diagnosed and provided access to specialists, all panelists were “very” or “somewhat” satisfied with their HCP support. More time and more information from the specialists would increase satisfaction.

“*A confirmed diagnosis reduces anxiety: it is empowering to know the cause and be able to address the problem*.” (adult patient)

**Figure 1:**
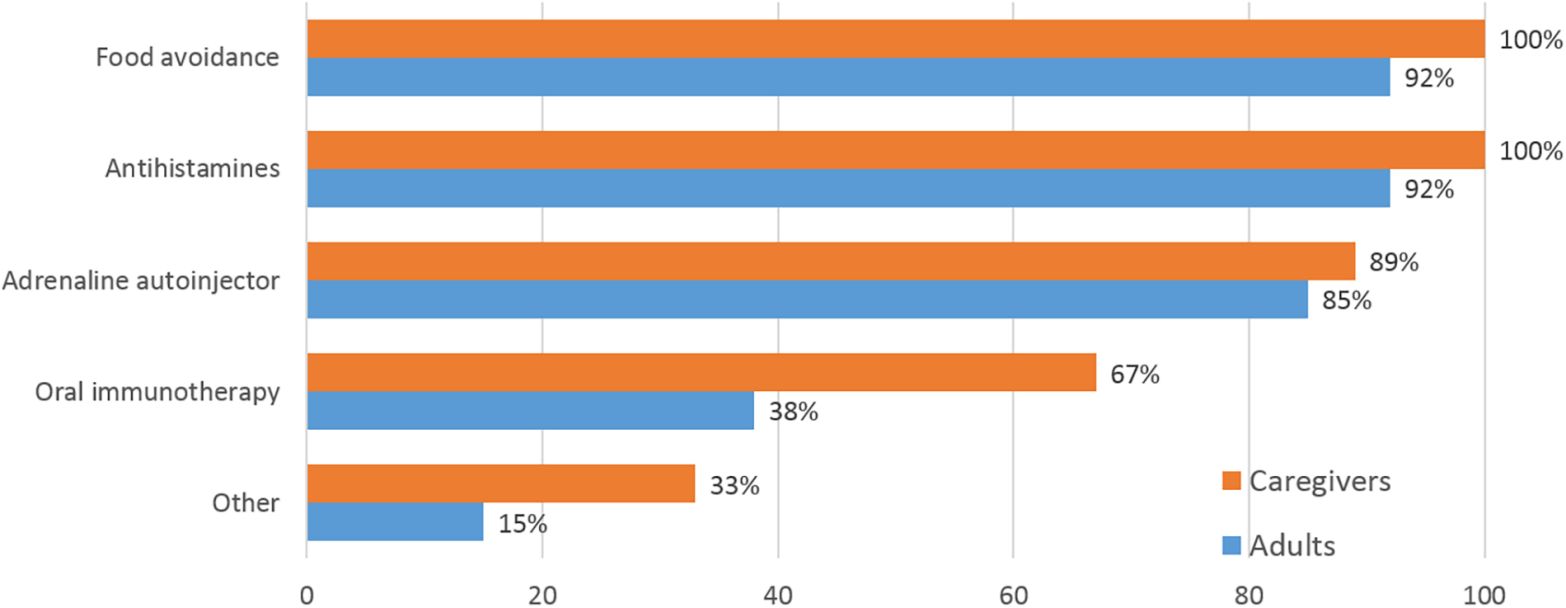
Treatment options for allergic reactions used by caregivers and adults. Multiple mentions were possible. “Other” included “*allergy shots to lower overall IgE*” (patient's words), adrenaline in emergency department, leukotriene, salbutamol or salmeterol + fluticasone.

Food avoidance and antihistamines were the most common treatments for food-allergic reactions in both groups (Figure 1). Adult patients and caregivers alike experienced food avoidance as a significant stress factor. Fewer adults than caregivers reported use of oral immunotherapy (OIT). All roundtable participants expressed worries about possible allergic reactions with OIT and several had unsuccessful experiences with the treatment at least initially (*My son's OIT was the hardest thing in my life in the past 10 years, but the “Extremely slow” OIT which is sometimes practiced in Japan finally worked well*.) Food avoidance measures were perceived as very successful by 67% and moderately successful by 33% of both groups of roundtable participants. Adrenaline [epinephrine] autoinjectors (AAIs) were kept at hand as an option for severe allergic reactions. Whereas adult panelists considered autoinjectors easy to use, even if they had never needed to inject, caregivers reported additional emotional stress thinking about the AAI, and low confidence in its use. “*I'm not totally confident in using the autoinjector, and I never have yet*.” Training in AAI use varied widely, from none at all to voluntary seminars for school teachers and school nurses run by physician or patient organizations. Several caregivers had trained their children themselves using expired AAIs and oranges.

**Figure 2:**
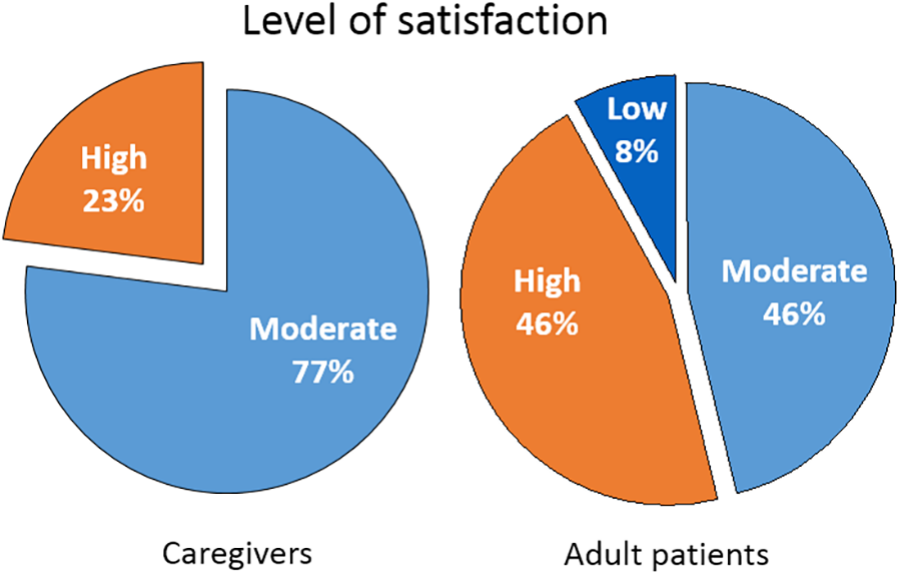
Level of satisfaction with current management of food allergies.

There were differences between caregivers and adults with food allergies in the expressed satisfaction with their current management (Figure 2). Adult patients presented a wider range, with greater percentages of highly satisfied but also of dissatisfied individuals. Caregivers expressed a need for psychological and emotional support which was often not covered well in their management.

**Figure 3:**
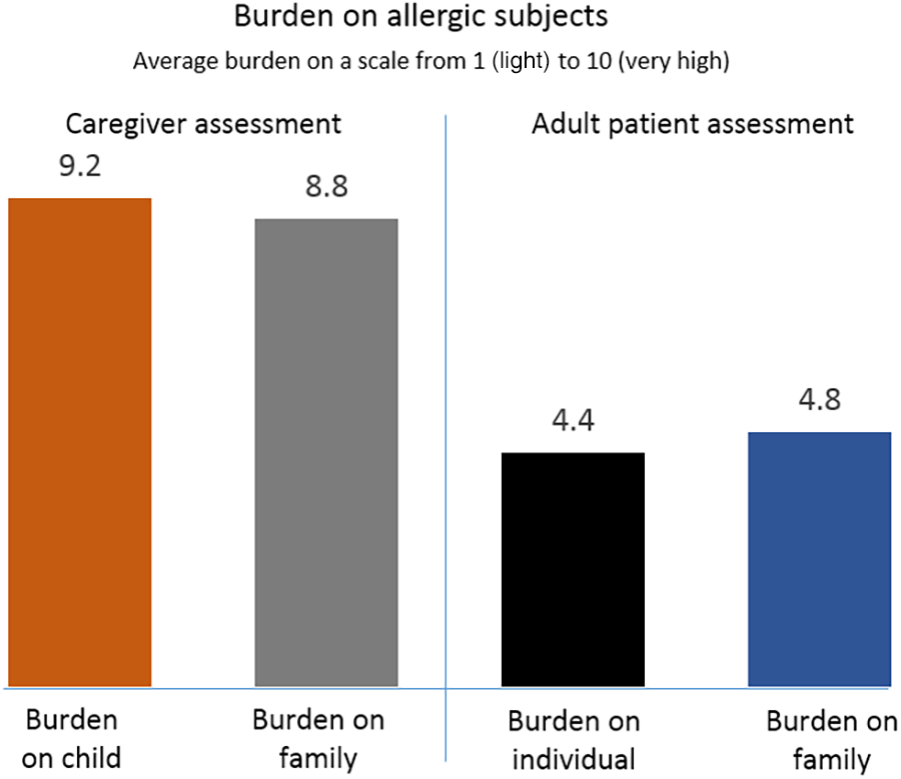
Burden of food allergy rated by caregivers and adult patients, respectively, on a scale from 1 (light) to 10 (heavy).

When rating the burden of food allergy on the lives of affected individuals and families, respectively, caregivers reported much greater burdens than adult patients (Figure 3). On a scale from 1 to 10, caregivers on average rated the burden on families at 8.8 and on children at 9.2. Adult patients on average rated the burden on families at 4.9 and that on themselves as individuals at 4.4. Adults recalled occasional bullying as children and the emotional stress when sensing parents'/caregivers' anxiety at hospital visits. Parents noted that children must be taught to be responsible for their own safety from an early age; to speak up and avoid risky situations; always carry an AAI; avoid suspicious food; suggest alternative foods to teachers, and other coping actions.

“*While the patient does much of the work, the family are on the journey too*” (adult patient)

“*Children have the greater burden because they will continue to deal with their food allergies, find a job, meet a partner, and have family*” (caregiver)

The pressure on families included siblings, who had in some cases been through traumatic situations, e.g., having to decide whether to administer adrenaline injections to younger siblings during an allergic reaction. Caregivers described an ever-present fear of inattention and of overlooking a risk factor for a severe reaction. “*It's torture watching your child have a reaction*.” The topic of fear was not brought up by the adult patients. Caregivers' worries increased with reduced control as children grew older and became more independent. Many, but not all, caregivers had adapted their working lives to the needs of their allergic children, reducing working hours, working from home, changing or even giving up employment to be near their dependents in case of an emergency.

“*My husband and I always make sure that at least one person is within three minutes of our child*”

In contrast, the prevailing attitude among the adult panelists was a determination not to let their food allergies get in the way of living their lives. In their working lives, adults working remotely and living on their own rarely thought about their allergy, although open-space workplaces, business meals, and travel might create unknown and challenging environments. Social life was affected in both groups of panelists, as in the words of one adult patient, “*just about every social event involves food*.”

Both adult patients and caregivers reported improved coping abilities with time, with caregivers often feeling less guilt than initially. All panelists agreed that despite improvements over time, wider society remains uninformed and awareness needs to improve in hospitality, schools, childcare and workplaces. In both roundtables inadequate or inconsistently regulated food labeling was considered an important issue, including for medicinal products which often contain food-derived ingredients, e.g., undisclosed lactose.

### Importance of information

**Figure 4:**
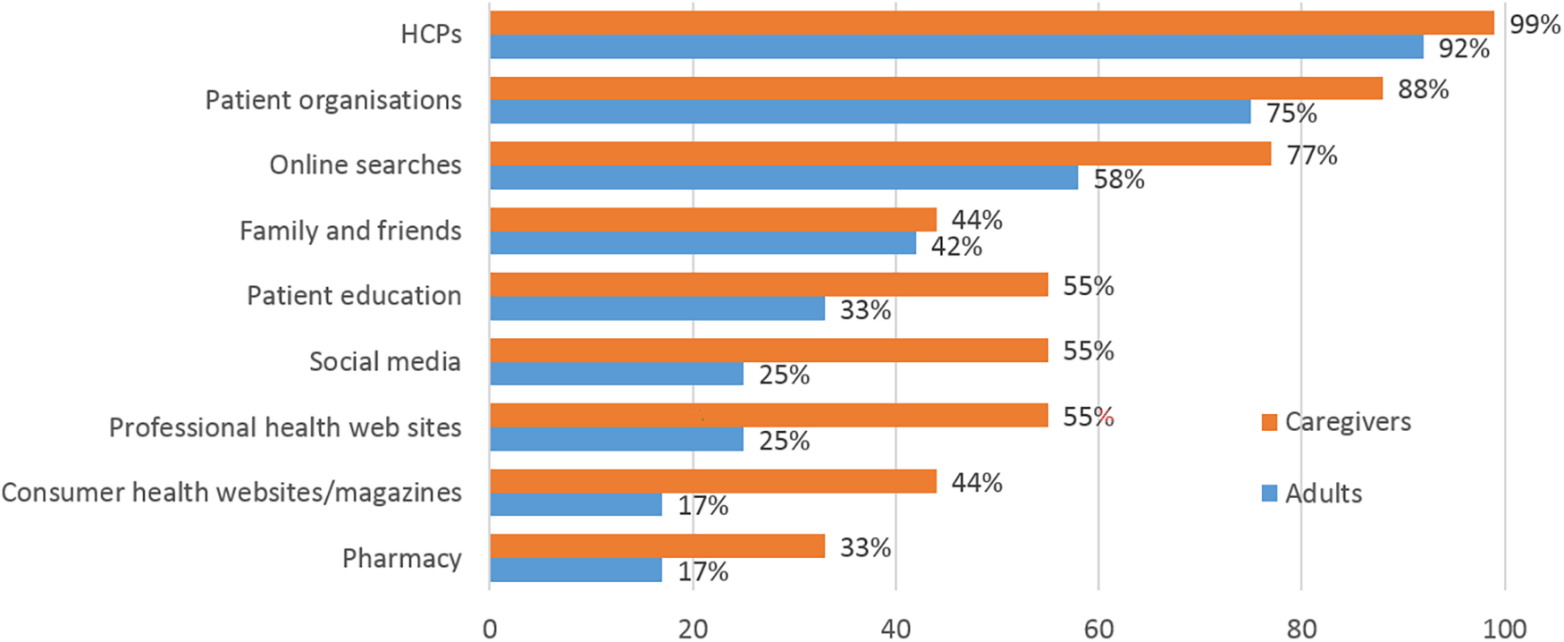
Most important sources of information about food allergy used by caregivers and adult patients, respectively. More than one mention was possible.

All roundtable participants actively informed themselves about food allergies, but there were differences between caregivers and adult patients in the choice of information sources (Figure 4). For both groups, HCPs and patient organizations followed by online searches were the most common sources of information. Fewer than half the adult patients used other sources of information. In contrast, most caregivers included social media and professional or consumer health web sites/magazines among their information sources. Different channels provided different information: HCPs and patient organizations were used for disease-related information or for managing reactions and symptoms, whereas social media were considered helpful for tips on restaurants, packaged foods and similar. Caregivers valued social media for making it easy to find patient organizations and practical advice. In countries such as China, where patient organizations are less active or well known, some of the information needs were filled by chat groups which included physicians. In all countries, wider society was considered very uninformed about food allergies, although the situation was slowly improving.

Both groups of panelists underlined the need for trusted sources of information. Caregivers in particular perceived the internet as full of misinformation from “well-meaning non-experts”.

### Attitudes to current and hypothetical emerging treatments

Adult patients and caregivers differed in their attitudes towards unmet needs from treatments. While both groups were safety-conscious, adult patients put a greater emphasis on convenience (Table 1) and reducing risk of severe reactions. Caregivers prioritized the need for reduced severity of reactions and for eliminating fear of the worst among children and caregivers:

**Table 1:** Needs in regard to therapies for food allergy from adult patients and caregivers, respectively.

Adult patients	Caregivers
Reduce the risk of severe reaction from accidental exposure to allergen	Reduced severity of reactions and eliminated survival risk
Greater effectiveness than current options	Ultimate goal: to abolish severe reactions so that whatever children eat they would not fear the worst
Convenience: No more complex than current food-avoidance schemes	Reduce the number of allergic incidences
Few side effects/low risk	Safety is key: If a treatment carries risks, it must be 100% effective. For treatments without risk a lower effectiveness is acceptable
Accessibility

“*We wish for not having to live in fear in a situation that occurs at least 3 times a day*”

## Discussion

These structured discussions among adult people with food allergy, or caregivers of children with the condition, respectively, with expert HCP attendance, showed similar experiences as well as several notable differences between the two groups of participants. This information may help physicians as well as drug developers improve the lives of both groups in the future.

Many of the reported life experiences confirm what has been reported elsewhere for caregivers, families and individuals with food allergies: a negative impact of the condition on social and professional lives, psychosocial pressure on families and caregivers (14), and a limited understanding among wider society, reflected, e.g., in the low quality of food labeling globally.

One striking difference between the groups was the much higher burden of disease experienced by caregivers for children than by adult patients. On a scale from 1 to 10, caregiver assessments were close to 9 for themselves as well as their children, compared with estimates of around 5 on the same scale by the adult patients. As the two roundtables were conducted separately, direct comparisons are not possible and any reasons for the differences between the groups are speculative. However, adult patients' personal experience of living with the illness vs. caregivers' responsibility for vulnerable dependents may play a role. Coping abilities improved with time among caregivers, families and adults alike, similar to what has been reported in other studies (11).

Adults and caregivers used a different value system from that of HCPs when assessing the severity of their food allergies. This indicates an important need for a common language between experts and those affected by the condition. Caregivers and adult patients defined “severe” very pragmatically, considering the term applicable when they perceived a relevant risk of anaphylaxis. This is different from how HCPs and the medical field define severity of reactions (21–24). As has been noted, there is no harmonized severity scoring of acute allergic reactions to serve the needs of all stakeholders, including patients, caregivers, allergy HCPs, and the food industry (25).

Caregivers' expressed a strong sense of fear. This was not encountered in the adult discussion group, who may have learnt to keep their fear under control. The constant worry about children experiencing a fatal event may be only weakly based on facts, as fatal food anaphylactic reactions are very rare (26–28). Yet anaphylaxis remains a serious reaction that is usually rapid in onset and often associated with hospitalizations, particularly in younger children (27), which provides reasons for caregivers' apprehensiveness.

Adult patients may feel more comfortable with food avoidance measures or AAI use after several years of independent lives, whereas caregivers will feel responsible for their charges and not always be in a situation to observe and control what they eat and how they may react. Moreover, adult patients carry early childhood experiences of food allergy into adulthood, adapting their early learnings to later stages in life. It is difficult for caregivers to recognize the extent or magnitude of symptoms being experienced by the child. Their high need for counseling in their day-to-day allergy management is a sign of the worries and uncertainties felt by caregivers. Other studies have shown that parents of allergic children often accompany them in social situations beyond the age at which nonallergic children are accompanied (6).

Caregivers' uncertainty about AAI use suggests that little has changed in the decade since Jacobs et al. reported that only one-third of initial food-allergic reactions with symptoms of anaphylaxis were recognized and treated with adrenaline (29). This is in contrast to the adults with food allergies, who reported widespread familiarity with AAIs. In the discussion, the attendees demonstrated wide knowledge about their allergies. However, other recent studies have reported widespread underuse and uncertainty around autoinjector use among adults and there seems to be a large degree of variation between affected individuals (30–32).

Both groups of panelists underlined the need for trusted sources of information, and caregivers in particular were suspicious of information from non-professionals. The adults, with longer personal experience of their food allergies, used fewer sources of information. Studies have shown that inaccurate perception of reaction severity and allergens is associated with reduced QoL among caregivers (8). The information gap is not easily closed, however, as research suggests that many general practitioners may be underprepared to address the needs of caregivers with children with severe food allergies, due to insufficient knowledge of the condition (33).

The need for safety was evident also in the discussions of hypothetical new treatments and clinical trials. In particular, caregivers were not ready to reduce their commitment to a food-avoiding diet if a new treatment were to emerge, whereas adult patients would be interested in trying out new food. However, the roundtables captured initial attitudes and goals with potential new treatments and these are likely to develop and change during the treatment journey. Adequate counseling would clearly be necessary before and during a new therapy. Caregivers were motivated by the prospect of less fear in the future, and although the adult patients emphasized hope for a cure as a motivating factor, safety was a key concern in both groups.

The roundtable discussions provided some insights which point to specific actions, summarized in Table 2. Many are common to both groups and most involve coaching, trusted information and a supportive environment. None is rocket science, although a globally standardized, high-quality system of food labeling would require international co-operation which may be very difficult to implement.

**Table 2:** Desirable actions emerging from the insights in the roundtable discussions.

Adult patients	Caregivers
Supporting social environment to reduce social marginalization	Thorough autoinjector training
Access to adequate counseling and close monitoring when trying new therapy	Access to adequate counseling and close monitoring when trying new therapy
Easy access to authoritative, trusted information	Easy access to authoritative, trusted information
Stronger support for the actions of patient organizations/patient advocacy groups	Stronger support for the actions of patient organizations/patient advocacy groups
Activities to increase awareness of food allergies and anaphylactic reactions in public places such as schools, restaurants…	Activities to increase awareness of food allergies and anaphylactic reactions in public places such as schools, restaurants…
Higher-quality, standardized food labeling world-wide	Higher-quality, standardized food labeling world-wide
Higher-quality, standardized labeling of medicines	Higher-quality, standardized labeling of medicines
	Increased government funding for research into etiology and therapeutic targets
	Fact checking tool to detect misinformation online

The format of an interactive, moderated online exchange was developed to overcome difficulties inherent in many methods of exploring patient and caregiver experiences. The platform and structure used in the current study have recently been used successfully in other conditions (19, 20). The virtual forum allowed busy individuals from four continents and a number of time zones to interact at their own convenience. Automated translation reduced the risk that the discussion was dominated by native or more fluent English speakers as each participant could use their own native language.

The study has limitations. To enable a roundtable discussion, the number of participants was limited, which may produce biased impressions of the situation in individual countries. A number of questions were structured, but the qualitative statements cannot be quantified. The granularity of the data obtained did not allow for analysis of, e.g., association between individual experiences and the allergic status of each participant, changes in patients' experiences from childhood to adulthood, or detailed food-avoidance measures for each offending food. The scope for generalizations to wider groups of caregivers and adults with food allergies is limited by the selection of participants in both groups, who represented engaged individuals, many of whom were in close contact with patient organizations and acted as advocates. For caregivers, this may have led to a bias towards more anxious individuals. Being a virtual discussion, participants' views may have been affected by comments from their peers on the platform. All participants were adults and the views of affected children could not be captured other than indirectly. A further limitation was that this pilot project involved caregivers and adults in discussions on separate occasions, without direct exchange between the groups. An important next step would be to use the same format for an inclusive roundtable with all stakeholders.

In summary, this interactive exchange provided important insights into the attitudes and experiences of adults and caregivers for children with food allergies. There were instructive differences notably in fear and confidence in daily life with the illness, the severity of its impact, and in unmet needs for treatments, which will be valuable to inform targeted management activities as well as further research. Both groups in addition confirmed the clear need for improved and widely available trusted sources of information, and for resources and training programs for potential new therapies. This opens opportunities to engage in digital solutions to meet these gaps for patients world-wide.

## Data Availability

The datasets presented in this article are not readily available because the full datasets (meeting transcripts) for this study are on file with Novartis. Requests to access the datasets should be directed to flavia.perna@novartis.com.

## References

[1] Nwaru BI Hickstein L Panesar SS Muraro A Werfel T Cardona V The epidemiology of food allergy in Europe: a systematic review and meta-analysis. Allergy. (2014) 69(1):62–75. 10.1111/all.12305 24205824

[2] Dunlop JH Keet CA. Epidemiology of food allergy. Immunol Allergy Clin North Am. (2018) 38(1):13–25. 10.1016/j.iac.2017.09.002 29132669

[3] Worm M Scherer K Köhli-Wiesner A Ruëff F Mahler V Lange L Food-induced anaphylaxis and cofactors - data from the anaphylaxis registry. Allergol Sel. (2017) 1(1):21–7. 10.5414/ALX01401E PMC6039999 30402598

[4] Grabenhenrich LB Dölle S Moneret-Vautrin A Köhli A Lange L Spindler T Anaphylaxis in children and adolescents: the European anaphylaxis registry. J Allergy Clin Immunol. (2016) 137(4):1128–1137.e1. 10.1016/j.jaci.2015.11.015 26806049

[5] Akarsu A Ocak M Köken G Şahiner ÜM Soyer Ö Şekerel BE. Ige mediated food allergy in Turkey: different spectrum, similar outcome. Turk J Pediatr. (2021) 63(4):554. 10.24953/turkjped.2021.04.002 34449137

[6] Cummings AJ Knibb RC King RM Lucas JS. The psychosocial impact of food allergy and food hypersensitivity in children, adolescents and their families: a review: the psychosocial impact of food allergy. Allergy. (2010) 65(8):933–45. 10.1111/j.1398-9995.2010.02342.x 20180792

[7] Springston EE Smith B Shulruff J Pongracic J Holl J Gupta RS. Variations in quality of life among caregivers of food allergic children. Ann Allergy Asthma Immunol. (2010) 105(4):287–94. 10.1016/j.anai.2010.08.003 20934628

[8] Howe L Franxman T Teich E Greenhawt M. What affects quality of life among caregivers of food-allergic children? Ann Allergy Asthma Immunol. (2014) 113(1):69–74.e2. 10.1016/j.anai.2014.04.016 24950845

[9] Moen ØL Opheim E Trollvik A. Parents experiences raising a child with food allergy; a qualitative review. J Pediatr Nurs. (2019) 46:e52–63. 10.1016/j.pedn.2019.02.036 30857930

[10] Lau GY Patel N Umasunthar T Gore C Warner JO Hanna H Anxiety and stress in mothers of food-allergic children. Pediatr Allergy Immunol. (2014) 25(3):236–42. 10.1111/pai.12203 24750570

[11] DunnGalvin A Blumchen K Timmermans F Regent L Schnadt S Podestà M APPEAL-1: a multiple-country European survey assessing the psychosocial impact of peanut allergy. Allergy. (2020) 75(11):2899–908. 10.1111/all.14363 32400915 PMC7689848

[12] Alanne S Laitinen K Paavilainen E. Living ordinary family life with an allergic child-the mother’s perspective. J Pediatr Nurs. (2014) 29(6):679–87. 10.1016/j.pedn.2014.06.012 25089834

[13] Birdi G Cooke R Knibb R. Quality of life, stress, and mental health in parents of children with parentally diagnosed food allergy compared to medically diagnosed and healthy controls. J Allergy. (2016) 2016:1–7. 10.1155/2016/1497375 PMC4939330 27429624

[14] Feng C Kim JH. Beyond avoidance: the psychosocial impact of food allergies. Clin Rev Allergy Immunol. (2019) 57(1):74–82. 10.1007/s12016-018-8708-x 30171460

[15] DunnGalvin A de BlokFlokstra BMJ Burks AW Dubois AEJ Hourihane JO. Food allergy QoL questionnaire for children aged 0-12 years: content, construct, and cross-cultural validity. Clin Exp Allergy. (2008) 38(6):977–86. 10.1111/j.1365-2222.2008.02978.x 18435800

[16] Knibb RC Huissoon AP Baretto R Ekbote A Onyango-Odera S Screti C Development and validation of the anaphylaxis quality of life scale for adults. J Allergy Clin Immunol Pract. (2022) 10:1527–33.e3. 10.1016/j.jaip.2022.02.023 35259537

[17] Cao S Borro M Alonzi S Sindher S Nadeau K Chinthrajah RS. Improvement in health-related quality of life in food-allergic patients: a meta-analysis. J Allergy Clin Immunol Pract. (2021) 9(10):3705–14. 10.1016/j.jaip.2021.05.020 34089927 PMC8511199

[18] Herbert L Marchisotto MJ Vickery B. Patients’ perspectives and needs on novel food allergy treatments in the United States. Curr Treat Options Allergy. (2021) 8(1):9–20. 10.1007/s40521-020-00274-8 33520599 PMC7825384

[19] Brujic M Kruger P Todd J Barnes E Wuttke M Perna F Living with presbyopia: experiences from a virtual roundtable dialogue among impacted individuals and healthcare professionals. BMC Ophthalmol. (2022) 22(1):204. 10.1186/s12886-022-02432-9 35513787 PMC9074271

[20] Caffery B Petris R Hammitt KM Montecchi-Palmer M Haque S Malkowski JP Patient perspectives on dry eye disease and chronic ocular surface pain: insights from a virtual community-moderated dialogue. Eur J Ophthalmol. (2022):11206721221125264. 10.1177/11206721221125263 36071618

[21] Chinthrajah RS Jones SM Kim EH Sicherer SH Shreffler W Lanser BJ Updating the CoFAR grading scale for systemic allergic reactions in food allergy. J Allergy Clin Immunol. (2022) 149(6):2166–2170.e1. 10.1016/j.jaci.2021.12.789 35026206 PMC9177543

[22] Arasi S Nurmatov U Dunn-Galvin A Daher S Roberts G Turner PJ Consensus on definition of food allergy severity (DEFASE) an integrated mixed methods systematic review. World Allergy Organ J. (2021) 14(3):100503. 10.1016/j.waojou.2020.100503 33767801 PMC7966874

[23] Dribin TE Schnadower D Spergel JM Campbell RL Shaker M Neuman MI Severity grading system for acute allergic reactions: a multidisciplinary Delphi study. J Allergy Clin Immunol. (2021) 148(1):173–81. 10.1016/j.jaci.2021.01.003 33476673 PMC8273088

[24] Cardona V Ansotegui IJ Ebisawa M El-Gamal Y Fernandez Rivas M Fineman S World allergy organization anaphylaxis guidance 2020. World Allergy Organ J. (2020) 13(10):100472. 10.1016/j.waojou.2020.100472 33204386 PMC7607509

[25] Muraro A Fernandez-Rivas M Beyer K Cardona V Clark A Eller E The urgent need for a harmonized severity scoring system for acute allergic reactions. Allergy. (2018) 73(9):1792–800. 10.1111/all.13408 29331045

[26] Umasunthar T Leonardi-Bee J Hodes M Turner PJ Gore C Habibi P Incidence of fatal food anaphylaxis in people with food allergy: a systematic review and meta-analysis. Clin Exp Allergy. (2013) 43(12):1333–41. 10.1111/cea.12211 24118190 PMC4165304

[27] Baseggio Conrado A Ierodiakonou D Gowland MH Boyle RJ Turner PJ. Food anaphylaxis in the United Kingdom: analysis of national data, 1998-2018. Br Med J. (2021) 372:n251. 10.1136/bmj.n251 33597169 PMC7885259

[28] Turner PJ Jerschow E Umasunthar T Lin R Campbell DE Boyle RJ. Fatal anaphylaxis: mortality rate and risk factors. J Allergy Clin Immunol Pract. (2017) 5(5):1169–78. 10.1016/j.jaip.2017.06.031 28888247 PMC5589409

[29] Jacobs TS Greenhawt MJ Hauswirth D Mitchell L Green TD. A survey study of index food-related allergic reactions and anaphylaxis management: a survey study of anaphylaxis management. Pediatr Allergy Immunol. (2012) 23(6):582–9. 10.1111/j.1399-3038.2012.01315.x 22625658

[30] Umasunthar T Procktor A Hodes M Smith JG Gore C Cox HE Patients’ ability to treat anaphylaxis using adrenaline autoinjectors: a randomized controlled trial. Allergy. (2015) 70(7):855–63. 10.1111/all.12628 25850463 PMC4654245

[31] Prince BT Mikhail I Stukus DR. Underuse of epinephrine for the treatment of anaphylaxis: missed opportunities. J Asthma Allergy. (2018) 11:143–51. 10.2147/JAA.S159400 29950873 PMC6016581

[32] Turner PJ DunnGalvin A Hourihane JO. The emperor has no symptoms: the risks of a blanket approach to using epinephrine autoinjectors for all allergic reactions. J Allergy Clin Immunol Pract. (2016) 4(6):1143–6. 10.1016/j.jaip.2016.05.005 27283056 PMC5123619

[33] Broome-Stone SB. The psychosocial impact of life-threatening childhood food allergies. Pediatr Nurs. (2012) 38(6):327–30. 23362632

