# Supplementary material for: Living with food allergies: the experiences of adult patients and caregivers
Source: Front Allergy. 2023 Nov 6;4:1272851. doi: 10.3389/falgy.2023.1272851 (PMC10658712; doi:10.3389/falgy.2023.1272851)
Supplement: Supplementary file 1 [file Table1.docx]

## Appendix

## Living with Food Allergy

Please briefly describe what living with food allergy is like for you, your child and your family on a daily basis.

How would you rate the impact your or your child’s food allergy has on their life on a scale of 1-10?

How would you rate the impact your or your child’s food allergy has on your life on a scale of 1-10?

What foods are you or your child allergic to?

How severe do you rate your or your child’s food allergy to be?

Did your physician discuss with you the various levels of food allergic reactions and whether a past reaction may or may not be a predictor of future reactions?

Do your or your child have other health conditions as well as food allergies (e.g. asthma, atopic dermatitis)? If so please let us know what they are if you are happy to do so.

What signs or symptoms do your or your child experience when you/they eat a food that you/they are allergic to?

How often do your or your child accidentally eat a food you are allergic to and what do you do when this happens? How often has this happened in the past 6 months or 1 year?

Please tell us about your or your child’s worst allergic reaction to a food you/they have eaten. How did you react and did you change anything in your approach to the food allergy? Have your or your child ever experienced anaphylaxis and if so what symptoms did you/they experience?

In what ways does the food allergy impact school / work / social life both physically and emotionally?

In what ways does the food allergy impact you and your family’s life both physically and emotionally?

## Pre-diagnosis and diagnosis of food allergy

Prior to diagnosis, what signs and symptoms of food allergy did you first notice and how long had you or the child had them? How old were your or your child at the time?

What prompted your first visit to a health specialist for the food allergy?

Which health specialist did you first see? Did you need a referral to an allergy specialist from a GP (primary care doctor)?

Which health specialist first diagnosed the food allergy?

How satisfied were you with your interaction with the health specialist at diagnosis? How could this interaction be improved?

## Treatment and management of food allergy

What types of food allergy testing did your or your child undergo prior to diagnosis and what testing have you/they undergone since then?

What treatment options were you first offered immediately after diagnosis and what else have you used since then?

Do your or your child carry an adrenaline autoinjector (Epipen) if they are available in your country? If so, how many times have you used it? If your child has used it, did you both feel confident in using it, and if not, please explain why?

Do you ever treat the allergic reactions with over-the-counter oral antihistamines? If so, why would this medication be chosen instead of using the adrenaline autoinjector (Epipen) if they are available in your country?

Which health specialist do you currently see for the food allergy, if any? How often do you see them and if you do not see one, why not or what has stopped you from doing so?

How satisfied are you overall with the quality of your interaction, including allocated time, with your food allergy health specialist(s)?

Have your or your child outgrown any food allergies since you/they were young?

If your or your child was prescribed an EpiPen or autoinjector, how did the physician characterize when the EpiPen should be used? Did he/she indicate it should be used for more severe reactions or whenever your or your ingests a food allergen and experiences any level of reaction?

## Needs along your food allergy journey

How has having a food allergy affected you at work / your child at school? For example do you think shool staff are knowledgeable and do they do anything to protect your child?

Do you think your friends/ family understand food allergies and are they supportive? Is there anything you do differently because of the food allergy?

Do you or your child use any digital tools to help you manage their food allergy, such as apps, wearable devices, web-based tools etc? If so, how useful do you find them and which one do you use the most?

What do you believe are the most important physical and emotional needs that are not being met in the current management of the food allergy? What is the most important issue your or your child face? What is the most important issue (i.e. what would help relieve your fear on a daily basis)?

What sources of information do you and your child use for their food allergy?

Are you a member of a food allergy patient support group? If so, in what ways do they offer you support?

What extra financial (eg particular foods, special diets, nutrition counseling etc) and time costs are associated with the food allergy? Please be as specific as possible.

How successful do you feel avoidance of foods that you or your child are allergic to is?

What challenges does avoidance of food involve for your or your child, and for other family/friends both in and out of the home? How difficult is it to adhere to and how does it affect/limit your and your child’s life?

What would your main goal be when trying a new treatment for food allergy?
